# Osteosarcoma: Accelerating Progress Makes for a Hopeful Future

**DOI:** 10.3389/fonc.2018.00004

**Published:** 2018-01-26

**Authors:** Amanda J. Saraf, Joelle M. Fenger, Ryan D. Roberts

**Affiliations:** ^1^Pediatric Hematology, Oncology, and BMT, Nationwide Children’s Hospital, Columbus, OH, United States; ^2^College of Veterinary Medicine, The Ohio State University, Columbus, OH, United States; ^3^Research Institute at Nationwide Children’s Hospital, Columbus, OH, United States

**Keywords:** osteosarcoma, drug development, metastasis, resistance, chemotherapy, immunotherapy of cancer, pediatric oncology

## Abstract

Patients who develop osteosarcoma in 2017 receive treatment that remains essentially unchanged since the 1970s. Outcomes likewise remain largely unimproved. Large, collaborative, multinational efforts to improve therapy have evaluated strategies leveraging both cytotoxic intensification and immunomodulatory agents. While these have confirmed our capacity to conduct such trials, results have proved largely disappointing. This has motivated efforts to focus on the basic biology of osteosarcoma, where understanding remains poor but has improved significantly. Recent advances have identified characteristic genetic features of osteosarcoma, including profound chromosomal disruption, marked patient-patient heterogeneity, and a paucity of recurrent mutations. Analyses suggest genesis in early catastrophic genetic events, although the nature of the inciting events remains unclear. While p53 and Rb inactivation occurs in most osteosarcomas, the landscape of associated driver mutations has proved extensive. Few mutations recur with high frequency, though patterns continue to emerge that suggest recurrent alterations within specific pathways. Biological pathways implicated in osteosarcoma biology through genetic and other preclinical studies include PI3K/mTOR, WNT/βcatenin, TGFβ, RANKL/NF-κB, and IGF. Unfortunately, clinical studies evaluating targeted agents have to date yielded disappointing results, as have studies examining modern immunotherapeutics. It remains unclear whether this pattern of clinical failures exposes inadequacies of our preclinical models, unrealistic expectations for single-agent responses in heavily pretreated patients, or biology less relevant than suggested. Nearly all patients who succumb to osteosarcoma develop lung metastases, which exhibit marked chemoresistance. Much scientific effort has recently sought to enhance our mechanistic understanding of metastasis biology. This research has potential to reveal novel targets for preventing and treating metastasis and for uncovering key vulnerabilities of osteosarcoma cells. Efforts to implement drug development strategies that leverage clinical studies in veterinary patients have potential to accelerate the translation of novel experimental regimens toward human studies. These could reduce costs and development timelines, prioritize agents, and refine regimens prior to human clinical trials. The rise of philanthropic groups focused on osteosarcoma has enhanced cross-disciplinary and cross-institutional focus and provided much needed resources. Transformative new therapies will likely arise from collaborative, interdisciplinary efforts that extend our understanding of osteosarcoma’s most basic inner workings.

## Introduction

Children diagnosed with cancer today benefit from years of incremental advances in therapy that have reduced childhood cancer mortality by over 50% since the mid-1970s (1). Beyond the impacts strictly on survival, the employment of risk stratification and other therapeutic advances have also brought decreases in secondary late effects, such as secondary malignancy (2) and chemotherapy-related heart and lung disease (3).

Children and adolescents diagnosed with osteosarcoma have not experienced similar advances. Those diagnosed with osteosarcoma in 2017 receive medical therapies that remain essentially unchanged since the introduction of MAP (methotrexate, doxorubicin, and cisplatin) in the late 1970s (4). Outcomes have likewise improved very little since the advent of chemotherapy. The 5-year overall survival remains about 60% and stagnant over the last five decades (5). Multiple efforts to improve therapeutic efficacy, including several cooperative international clinical trials, have not identified more effective or less toxic regimens, despite efforts to intensify treatment and to modulate immune responses (6–10).

Those who develop metastatic disease continue to experience dismal outcomes; development of pulmonary metastasis heralds the onset of chemoresistance and a pivot toward more experimental therapies, given the lack of hope offered by currently established therapies. Prognosis remains poor whether metastases develop prior to diagnosis or long after completing therapy (11). 5-year overall survival rates remain about 20%, despite numerous attempts to improve therapy through intensification (12–14).

Patients diagnosed today clearly benefit from advances made in both the surgical treatment of disease and in the provision of supportive care. Limb-sparing methods pioneered in canine patients with osteosarcoma (15) have become standard of care. Nearly 85% of patients undergoing resection since the year 2000 have been able to keep their limbs (5). We should note that patients undergoing treatment today clearly have experiences different from those of patients in previous decades. Numerous advances in emergency care, bone marrow support, antiemetics, and companion protective regimens reduce morbidities associated with treatment and make that treatment far more tolerable.

In this review, we briefly outline several emerging discoveries that advance the known biology of osteosarcoma as well as several ongoing initiatives that offer hope for the future. We highlight and discuss several aspects of osteosarcoma biology that suggest opportunities where improvements in our understanding of the underlying biology might lead to meaningful therapeutic advances. These include: the molecular biology of osteosarcomagenesis, mechanisms that drive pulmonary metastasis, and a rapidly evolving understanding of the interface between immunology and oncology. We will also highlight two broad initiatives that promise to enhance our ability to affect change, namely the rapidly growing availability of well-characterized models of disease and the organization of advocacy groups that push science forward and fill much-needed philanthropic niches to make that science possible.

## Molecular Biology of Osteosarcoma

Transformative advances in the treatment of osteosarcoma will likely emerge from insights into the mechanistic biology that underlies the disease. Large-scale efforts to characterize the genomic landscape of osteosarcoma have revealed a genome characterized by marked inter- and intra-tumoral heterogeneity, chains of complex chromosomal rearrangements, widespread gene copy number alterations, and localized regions of hypermutation (16–18). Unbiased genome-wide screening techniques have sought drivers of osteosarcoma genesis and metastasis (19). Larger, well-annotated datasets coupled with large-scale characterization of genetic, epigenetic, and transcriptional changes generated through the TARGET (20) initiative now provide vast collections of data that can accelerate the generation and testing of novel hypotheses.

These analyses support several long-suspected hypotheses. They demonstrate near-universal loss of p53 through a number of mechanisms and frequent alterations of Rb (16, 18, 19). Increasing numbers of analyses suggest recurrent alterations in the PI3K/mTOR pathway, including loss of PTEN (18, 19, 21). Efforts have uncovered few other genetic mutations that recur with high frequency.

Smaller-scale hypothesis-driven investigations have suggested roles for other pathways in the progression of osteosarcoma. WNT/β-catenin pathway activation appears to drive some characteristics of proliferation and early metastasis (22–24). Aberrant expression of the ΔNp63 pathway appears very frequently in osteosarcoma and may drive key elements of osteosarcomagenesis and metastasis, likely through the production of IL6 and CXCL8 (25).

Tumor–host interactions appear critical to the development of osteosarcoma. Some of these microenvironmental factors have been identified, including stromal-derived TGFβ (26) and IL6 (27). Activation of NF-κB through RANK/RANKL may play a role in development and progression (28, 29), as may stimulation by IGF family members (30). Recent studies have suggested significant antitumor activity can result from blockade of these stromal-derived signals (31).

One of the most clinically relevant applications of this molecular biology work comes from identification of pathways responsible for chemoresistance. A number of studies hint at mechanisms of resistance, most notably that arising from activation of the PI3K/Akt/mTOR pathway (18, 21, 32, 33). Whether targeting this pathway (or others) can restore sensitivity to resistant clones in a clinical setting remains an unknown but important question.

Unfortunately, among all preclinical work suggesting efficacy with targeting of these pathways, few invoke more than growth delays, making their potential clinical utility suspect. This could result because (1) these do not represent major driver pathways of the disease, (2) redundancies and escape/resistance mechanisms exist, which render these targeting methodologies ineffective, or (3) these pathways drive biology in a subset of tumor cells within a heterogeneous tumor. Regardless of the reason, advances will not likely come from single-pathway targeting, but from identifying synthetic lethalities resulting from specific combinations.

Recent reports detail intriguing patterns of gene copy number variation in OS, suggesting that the chaotic chromosomal rearrangements of OS might lead naturally to amplification of oncogenes or loss of tumor suppressors that drive malignant progression (34). These reports suggest a precision oncology strategy, where alterations identify subgroups of tumors with specific targetable vulnerabilities. While these nascent concepts remain relatively unproven, they can readily be tested. Such novel approaches have potential to revolutionize care. Preclinical evaluations are actively ongoing.

## Osteosarcoma Metastasis

Metastasis remains the most important fatal complication of osteosarcoma. Among those who develop metastasis, less than 1 in 5 survive (11). A therapy preventing the emergence of OS lung metastases or facilitating the treatment of those metastatic lesions could potentially save more than 70% of the lives currently lost to the disease (11, 35, 36). This would represent the single most significant improvement in outcome for OS since the advent of chemotherapy in the 1960s. Our limited understanding of the biology, which drives the spread of OS from primary bony sites into lung tissue limits our ability to accomplish this (37).

Several of the molecular mechanisms identified as driver pathways in OS clearly play key roles in the metastatic cascade. WNT/β-catenin pathway activation likely facilitates early steps, including invasion, chemotaxis, and extravasation (22, 24), and some studies suggest activity of agents that block this pathway (23). Notch appears to have similar activity, which one can target using γ-secretase inhibitors (38). OS cells express high levels of ezrin, which links the actin cytoskeleton to the extracellular matrix and serves as a scaffold for PI3K/Akt signaling (39). Inhibitors of ezrin can block invasion in OS cells and inhibit metastasis (40, 41).

ΔNp63-derived IL6 and CXCL8 produced by tumor cells clearly play key roles in lung colonization. Gene manipulation studies suggest that targeting these processes could block metastasis (25, 42). Clinical agents exist that could target these pathways. Lung colonization likely requires a number of lung-specific metabolic adaptations, which could be targetable. A few labs have begun to focus on identifying these metabolic sensitivities, such as metastasis-associated upregulation of gp78 (43, 44), protein kinase C activation (45), or mTOR pathway activation (32).

The relatively slow pace for identification of specific mechanisms that effect metastasis highlights some of the challenges inherent to this type of research. Several of the most recent developments, however, identify mechanisms that not only appear to represent real metastatic dependencies but can also be targeted using existing therapeutics (46). While many pathways critically important to metastasis remain unidentified, many hope that ongoing evaluation of these novel approaches will offer new therapeutic opportunities that target disease progression in novel ways.

## Immuno-Oncology of Osteosarcoma

Some have suggested that the genomic complexity of osteosarcoma may divulge a sensitivity to immunotherapy (47). Efforts to harness the immune system in the fight against osteosarcoma trace back to reports of improved outcomes in patients with severe wound infections and to some intriguingly positive outcomes observed in patients treated with interferon alone (48).

Unfortunately, this hope has not yet translated into robust clinical benefit. The EURAMOS-1 trial randomized a large number of good histologic responders to maintenance with or without interferon α2b, but failed to demonstrate benefit (6). The other large international trial evaluated the addition of muramyl tripeptide to standard of care, on the premise that the drug would stimulate macrophages to aid in tumor destruction. While this study built on a significant body of preclinical data, including clinical trials in dogs that showed increased survival and decreased metastatic disease (49), the trial was plagued with statistical challenges (36). These data eventually showed some definitive benefit for muramyl tripeptide (10), but not until business realities forced decisions that halted development within the United States, severely limiting availability.

More recent efforts seek to capitalize on the successes seen with immune checkpoint inhibitors in other cancers by extending those regimens to patients with osteosarcoma. Expression of checkpoint molecules like PD-L1 on osteosarcoma cells correlates with metastasis and decreased survival (50, 51), and murine studies suggest at least some activity of checkpoint inhibitors in osteosarcoma (52). Results in human trials using largely single-agent checkpoint inhibitor therapies have, to date, proved disappointing (53, 54). Some suggest that the relatively lower mutational burden seen in osteosarcoma relative to the hypermutant tumors that exhibit predictable responses to checkpoint inhibition may generate a neoantigen load inadequate to drive adoptive immune responses (55). Many retain hope, however, that improved understanding of the immunosuppressive microenvironment within osteosarcoma tumors will facilitate intelligent combinations of therapies that will unleash the power of immune oncology approaches as effective treatments for the disease.

## Improving Models: Patient-Derived Xenografts (PDX) and Comparative Oncology

Investigators worldwide have generated, developed, and characterized an ever-growing number of models that researchers can leverage in the study of osteosarcoma. Numerous cell lines (56) provide models that investigators can manipulate easily in the laboratory. Genetically engineered murine models allow for investigation into osteosarcoma pathogenesis and progression (57–60). One can combine these models in a number of ways to answer questions about causation and mechanisms of progression using rigorous scientific methodologies.

Some question how well these models represent the true intrinsic biology of the human disease (61, 62). Cell culture imposes widespread genetic changes and loss of phenotypic heterogeneity that diverge from the characteristics of the original tumor (63). Osteosarcoma cells maintained in culture demonstrate significant changes in phenotype over time (64). Many hope that ever-expanding libraries of PDX will address these shortcomings, and early evidence seems promising (65, 66).

Evaluating biology and novel therapeutic strategies using selected libraries of PDX tumors can overcome limitations inherent to more common traditional approaches (67, 68). While the cell lines and models most widely employed across the literature represent a relatively narrow group of OS tumor subtypes, the integration of bioinformatics into the PDX research pipeline facilitates both the selection of relevant models and the identification of shared characteristics that can identify biomarkers and mechanisms of response. These associated datasets allow investigators to select models either representative of the spectrum of human disease or sharing particular characteristics which might predict response to a particular regimen.

Patient-derived xenografts models poorly address several increasingly relevant aspects of tumor biology. Most of these limitations stem from the need to utilize immunodeficient mice in the passaging of human tumors, making study of immune-based therapies and other tumor–host interactions difficult. The emergence of systems and science around comparative oncology has great potential to address these shortcomings (69, 70). This comparative approach leverages clinical trials performed in veterinary settings, enrolling companion animals (pet dogs that have spontaneously developed osteosarcoma), to identify novel agents and combinations with activity (71, 72).

Biologically, canine osteosarcomas appear nearly indistinguishable from the human disease. The study of disease in one species will likely benefit the other, and *vice versa* (42, 73–76). Dysregulation of candidate genes implicated in the pathogenesis of osteosarcoma have been identified in both species, including mutations in the tumor suppressor genes PTEN, p53, and RB1 and alterations in the oncogenes MET and STAT3 (71).

The study of naturally occurring osteosarcoma in pet dogs has particular relevance to the study of immune-based therapies. In dogs, disease develops in the context of an intact immune system and accurately models the complex biology of the cancer microenvironment (77). Recognizing this unique potential, NCI recently released requests for application targeting studies that evaluate immune-based therapies in canines undergoing veterinary treatment for cancer. Many hope that broader implementation of integrated drug development (using canine patients) will reduce regulatory burdens, financial costs, and clinical timelines in ways that accelerate the identification of more effective approaches (69).

While metastasis and development of resistance represent the biological processes most important to clinical outcomes, these are poorly modeled in most of the assays routinely used for preclinical development of novel therapeutics. Ideally, preclinical investigation will reveal specific, actionable targets that lead to an initial focused screening. Models exist, which can facilitate validation of these candidates, as well as more broad-based screening of small libraries, though doing so remains cumbersome and comes with a number of biological caveats (37). The increasing organization of canine comparative oncology groups creates capacity for the study of therapies that affect metastasis and resistance using models that more closely recapitulate clinically relevant biology on time courses many fold faster than in humans.

## Philanthropy and Advocacy

The emergence of innovative and organized philanthropic and advocacy groups with strong disease focus has catalyzed movement around several recent initiatives. Their engagement provides not only badly needed funding but their relative detachment from the worlds of academia and industry allows them to foster collaborations and address systemic needs that lack incentive within those communities. Not content to limit their roles to advocacy and education, these groups have overseen the organization and annotation of large tissue banks, including osteosarcoma (78, 79). Their support proved instrumental in inclusion of osteosarcoma in NCI’s TARGET initiative. Analyses performed independently by some lay groups have intensified investigations exploring the establishment of biologically distinct subgroups of osteosarcoma. These efforts unquestionably accelerate discovery with potential to drive the development of much-needed novel therapies.

## Conclusion

**Figure 1:**
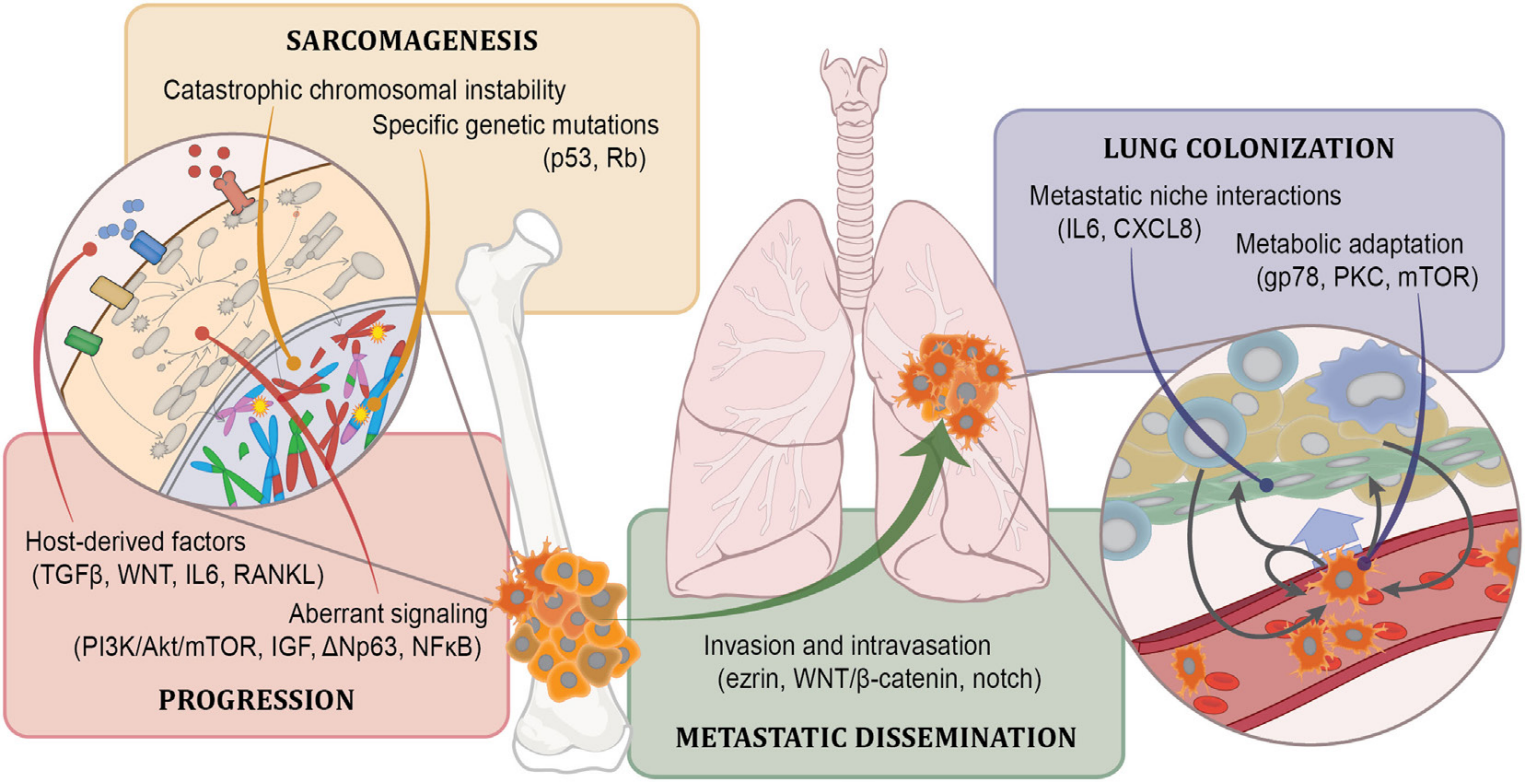
Known osteosarcoma biology. Our current understanding defines osteosarcoma in terms of the involvement of particular pathways in specific malignancy-associated processes. For some of these, the mechanisms by which these pathways affect those processes is known in detail (such as for p53 loss in sarcomagenesis). For most, however, the mechanisms through which they affect malignancy are known more generally. The degree to which an involved pathway presents opportunity for targeting depends both on the specific process it mediates, the biochemical properties of the pathway, and the stage of development in which it operates. For instance, it might not make sense to target metastatic dissemination if the process has already occurred when patients are diagnosed. Alternatively, tumors may remain addicted to core processes important for sarcomagenesis even after relapse and metastasis, making them attractive targets, but the biochemistry of those pathways may prove difficult to manipulate. Vast opportunities remain within each of these realms to both identify relevant biology and to clarify the role these factors play in the development and progression of osteosarcoma.

Development of novel therapies for osteosarcoma has suffered a discouraging past. Our maturing understanding of some fundamental aspects of osteosarcoma biology (Figure 1), coupled with intense focus on the mechanisms that drive metastasis and tumor heterogeneity, supported by the growing availability of large, readily available, clinically annotated data sets has directly supported the development of several important scientific and conceptual advances. Growing collections of tumor models that more faithfully recapitulate the human disease and the maturing of comparative oncology programs provide ever-more-efficient systems for rapid preclinical evaluation. The engagement of philanthropic and advocacy groups personally vested in the cause can only accelerate timelines and motivate ongoing development.

This confluence of scientific and supportive developments leads many physicians and scientists to a hopeful anticipation that transformative discoveries might lie upon a very near horizon. One might argue that the future, in fact, looks very bright. With our patients in mind, we hope that this quickening pace of discovery will translate into real clinical advances for patients who need better options now.

## Author Contributions

AS, JF, and RR all contributed to the design, drafting, revision, and final approval of this manuscript.

## Conflict of Interest Statement

The authors declare that the research was conducted in the absence of any commercial or financial relationships that could be construed as a potential conflict of interest.
